# Heterogeneity of Gestational Diabetes and Risk for Adverse Pregnancy Outcome: A Cohort Study

**DOI:** 10.1210/clinem/dgae754

**Published:** 2024-10-24

**Authors:** Yixin Gong, Qunhua Wang, Suyu Chen, Yujie Liu, Chenghua Li, Rong Kang, Jing Wang, Tian Wei, Qin Wang, Xianming Li, Sihui Luo, Jianping Weng, Xueying Zheng, Yu Ding

**Affiliations:** Department of Endocrinology, The First Affiliated Hospital of USTC, Division of Life Sciences and Medicine, University of Science and Technology of China, Hefei 230001, China; Department of Obstetrics and Gynecology, The First Affiliated Hospital of USTC, University of Science and Technology of China, Hefei 230001, China; Department of Obstetrics and Gynecology, The First Affiliated Hospital of USTC, University of Science and Technology of China, Hefei 230001, China; Department of Obstetrics and Gynecology, The First Affiliated Hospital of USTC, University of Science and Technology of China, Hefei 230001, China; Department of Cardiology, The First Affiliated Hospital of USTC, Division of Life Sciences and Medicine, University of Science and Technology of China, Hefei 230001, China; Department of Endocrinology, The First Affiliated Hospital of USTC, Division of Life Sciences and Medicine, University of Science and Technology of China, Hefei 230001, China; Department of Endocrinology, The First Affiliated Hospital of USTC, Division of Life Sciences and Medicine, University of Science and Technology of China, Hefei 230001, China; Department of Endocrinology, The First Affiliated Hospital of USTC, Division of Life Sciences and Medicine, University of Science and Technology of China, Hefei 230001, China; Department of Endocrinology, The First Affiliated Hospital of USTC, Division of Life Sciences and Medicine, University of Science and Technology of China, Hefei 230001, China; Department of Endocrinology, The First Affiliated Hospital of USTC, Division of Life Sciences and Medicine, University of Science and Technology of China, Hefei 230001, China; Department of Endocrinology, The First Affiliated Hospital of USTC, Division of Life Sciences and Medicine, University of Science and Technology of China, Hefei 230001, China; Department of Endocrinology, The First Affiliated Hospital of USTC, Division of Life Sciences and Medicine, University of Science and Technology of China, Hefei 230001, China; Department of Endocrinology, The First Affiliated Hospital of USTC, Division of Life Sciences and Medicine, University of Science and Technology of China, Hefei 230001, China; Department of Endocrinology, The First Affiliated Hospital of USTC, Division of Life Sciences and Medicine, University of Science and Technology of China, Hefei 230001, China

**Keywords:** gestational diabetes, heterogeneity, insulin resistance, pregnancy outcomes

## Abstract

**Context:**

Diabetes is increasingly recognized as a heterogeneous disease, with clinical characteristics and outcome risks varying across different phenotypes. Evidence on heterogeneity of gestational diabetes (GDM) is yet to be provided.

**Objective:**

To investigate the insulin physiology and pregnancy outcomes of GDM phenotypes characterized by fasting hyperglycemia or postload hyperglycemia.

**Methods:**

A total of 2050 women who underwent a 75-g oral glucose tolerance test were prospectively recruited and followed until delivery. Women were categorized into normoglycemia (NGT, n = 936), isolated impaired fasting glucose (gestational-IFG, n = 378), and isolated impaired postload glucose tolerance (gestational-IGT, n = 736) groups. Fasting blood samples at mid-pregnancy were collected to measure C-peptide and insulin concentrations. Homeostasis model assessment (HOMA) and quantitative insulin sensitivity check index (QUICKI) were used to evaluate insulin physiology. Maternal and neonatal outcomes were recorded.

**Results:**

Gestational-IFG had greater insulin resistance (HOMA-IR 3.11 vs 2.25, QUICKI-C-peptide 0.94 vs 1.03, both *P* < .01), and gestational-IGT had worse β-cell function (C-peptide 2.00 vs 2.26 ng/mL, *P* < .05), when compared to one another. Gestational-IFG was more strongly associated with excessive gestational weight gain (risk ratio [RR] 1.62; 95% CI, 1.18-2.23) and large-for-gestational-age infants (RR 1.45; 95% CI, 1.03-2.03) than gestational-IGT. The risk for neonatal brain injury was increased in gestational-IGT (RR 2.03; 95% CI, 1.04-4.09), but not in gestational-IFG (*P* = .439). Gestational-IGT showed a stronger association with the risk of preterm birth compared to gestational-IFG (RR 1.80; 95% CI, 1.02-3.36).

**Conclusion:**

GDM exhibits distinct insulin physiology profiles. Pregnancy outcome varies between each phenotype. These findings provide evidence on risk stratification and diverse strategies for the treatment of GDM.

Gestational diabetes mellitus (GDM) is a metabolic disorder during pregnancy and is related to an increased risk of adverse pregnancy outcomes. The etiology of diabetes involves pregnancy-induced insulin resistance and β-cell dysfunction. Previous studies have identified diabetes as a heterogeneous disease with subgroups of fasting hyperglycemia and postload hyperglycemia that involve distinct etiologies (1, 2). Furthermore, diabetes subgroups showed diverse clinical courses and different risk profiles for outcomes (3, 4). However, the approach to address the GDM heterogeneity and whether clinical outcome varies across subgroups remains unclear.

Explorations on the heterogeneity of GDM have shown inconsistency regarding insulin resistance, with some reporting greater insulin resistance for fasting hyperglycemia (5) while other reporting a similar level of homeostasis model assessment of insulin resistance (HOMA-IR) between fasting hyperglycemia and postload hyperglycemia subgroup (6). A population-based study linking GDM subtype to future type 2 diabetes (T2DM) concluded that women with isolated fasting hyperglycemia had higher risk of developing postpartum T2DM than those with isolated postload hyperglycemia (7). With regard to pregnancy outcome, previous studies generally showed higher risk of large-for-gestational-age (LGA) infants for women with GDM with fasting hyperglycemia than for those with postload hyperglycemia (5, 8-10), whereas women with postload hyperglycemia had greater risk of preterm birth compared to women with fasting hyperglycemia (5, 6, 11). However, evidence on other core outcomes for diabetes in pregnancy highlighted by international consensus (12), for example, trimester-specific hemoglobin A1c [HbA1c], gestational weight gain, preeclampsia, congenital malformations, has been scarce. Explorations with a well-designed cohort are therefore needed.

We conducted a prospective cohort of women screened for risk factors for GDM. We previously reported that GDM subtypes of fasting or postload hyperglycemia have distinct glucose profiles, with greater glucose excursion for women with postload hyperglycemia as estimated by a continuous glucose monitoring system (13). The current analysis aimed to estimate the insulin physiology and pregnancy outcomes of each GDM subtype. This exploration would provide a basis for risk stratification and strategies to prevent GDM and related adverse pregnancy outcomes.

## Methods

### Study Design and Population

This study was a prospective cohort of pregnant women who attended the First Affiliated Hospital of the University of Science and Technology of China between October 1, 2021, and January 1, 2024 (ChiCTR2000030972). Women who were pregnant between 4 and 27 weeks of gestation were recruited and screened for risk factor(s) for GDM (age at conception ≥ 35 years, body mass index [BMI] at conception ≥ 23.9 kg/m^2^, family history of diabetes, multiparity, polycystic ovary syndrome [PCOS], in vitro fertilization, history of hypertension or current systolic blood pressure [SBP] ≥ 140 mmHg and/or diastolic blood pressure [DBP] ≥ 90 mmHg, history of GDM). A 75-g oral glucose tolerance test (OGTT) was performed between 24 and 28 weeks gestation. The International Diabetes in Pregnancy Study Groups (IADPSG) recommendations were used for diagnosing GDM: fasting glucose ≥ 5.1 mmol/L (92 mg/dL), 1-hour postload glucose ≥ 10.0 mmol/L (180 mg/dL), or 2-hour postload glucose ≥ 8.5 mmol/L (153 mg/dL) (14). Those who met the criteria of GDM were invited to visit the intensive diabetes care unit. Lifestyle intervention and insulin treatment when deemed necessary after assessment by an endocrinologist were provided.

A total of 2050 women were enrolled and followed up until delivery. Standardized questionnaires were used to collect information on sociodemographic and lifestyle factors through face-to-face interviews (age, education, family history, reproductive history, smoking, and alcohol consumption). Body weight and height were measured with an Omron automatic height and weight machine. Seated SBP and DBP were measured via an Omron automated blood pressure monitor on a single occasion after the participants had rested for ≥ 10 minutes. Written informed consent was obtained.

Maternal plasma samples were collected at 4 visits: the first trimester (13 weeks and 6 days gestation or less, Visit 1), the second trimester (from 14 weeks to 27 weeks and 6 days gestation, Visit 2), the third trimester (from 28 weeks to 33 weeks and 6 days gestation, Visit 3), and the peri-delivery period (from 37 weeks to delivery, Visit 4). Plasma samples were analyzed within 4 hours of collection or stored at −80 °C for subsequent analysis. Fasting plasma glucose (FPG) was examined at Visit 1 in the first trimester (generally at 12 weeks gestation due to the context that the maternity file for prenatal care was built at 12 weeks gestation concomitant with blood tests). C-peptide (RRID: AB_3661822) and insulin (RRID: AB_3661823) were examined at Visit 2 via an auto electrochemiluminescence immunometric assay (AutoLumo A2000 Plus, China). HbA1c was examined with plasma sample at Visit 4.

The inclusion criteria of the current analysis were singleton pregnancy, gave birth to a live-born infant, and underwent a standard OGTT at 24 to 28 weeks gestation. The exclusion criteria were pregestational diabetes, both fasting and postload glucose levels exceeded thresholds, and incomplete OGTT. Comparisons were made across the 3 groups: (1) normal glucose tolerance (NGT group); (2) GDM characterized by isolated impaired fasting glucose (gestational-IFG group); and (3) GDM characterized by isolated impaired postload glucose tolerance (gestational-IGT group).

### Assessment of Insulin Sensitivity and β-cell Function

The severity of insulin resistance and β-cell function were evaluated using the HOMA method for insulin resistance (HOMA-IR), insulin sensitivity (HOMA-IS), and β-cell function (HOMA-B) based on fasting glucose and insulin levels with frozen plasma samples collected at clinic on Visit 2. The following formulas were used: HOMA-IR = fasting insulin concentration× FPG/22.5, HOMA-IS = 22.5/fasting insulin concentration × FPG, and HOMA-B = 20 × fasting insulin concentration/(FPG- 3.5). The degree of insulin sensitivity was also assessed by the quantitative insulin sensitivity check index (QUICKI) calculated with insulin (QUICKI-I) and C-peptide (QUICKI-CP): QUICKI-I = 1/(log[insulin] + log[FPG]), QUICKI-CP = 1/(log[C-peptide] + log[FPG]) (15).

### Outcomes

Pregnancy outcomes were recorded and stored on the Research Electronic Data Capture (REDCap) platform. Maternal outcomes included cesarean section, gestational hypertension, preeclampsia, and excessive gestational weight gain (GWG). Preeclampsia is defined as SBP ≥ 140 mmHg and/or DBP ≥ 90 mmHg accompanied by positive result of urinary proteinuria (≥1+) + or without proteinuria but accompanied by terminal organ disease. Neonatal outcomes included LGA (birth weight higher than the 90th percentile of gestational age), small for gestational age (SGA, birth weight lower than the 10th percentile of gestational age), low birth weight (LBW, birth weight < 2500 g), preterm birth (gestational age < 37 weeks at delivery), neonatal intensive care unit (NICU, admission to a higher-level neonatal care nursery > 24 hours during the initial hospitalization after birth), neonatal jaundice (a need for phototherapy or exchange transfusion), neonatal respiratory distress syndrome (NRDS), neonatal hypoxemia, neonatal brain injury (ICD-10 codes P11.0 to P11.2), and congenital heart defects (ICD-10 codes Q20 to Q28).

### Ethics Approval and Consent to Participate

This study was approved by the Ethics Committee of the First Affiliated Hospital of the University of Science and Technology of China (No.2021-KY-LS[241]) and was performed under the guidelines of the Helsinki Declaration. Writing informed consent was obtained from all the participating women.

### Statistical Analysis

Analyses were performed with R software (version 4.1.1). Continuous variables were summarized as means (95% CIs), and categorical variables were summarized as counts and proportions. To compare baseline characteristics and outcomes across groups, we used ANOVA test and chi-squared test where appropriate. Pairwise comparisons were made with *t* test. Log-binomial regression analysis was used to calculate adjusted risk ratios (RRs) and the respective 95% CIs for pregnancy outcomes adjusted for maternal age, education, and parity. Significance was set at two-sided *P* value < .05.

## Results

### Clinical Characteristics

**Figure 1.**
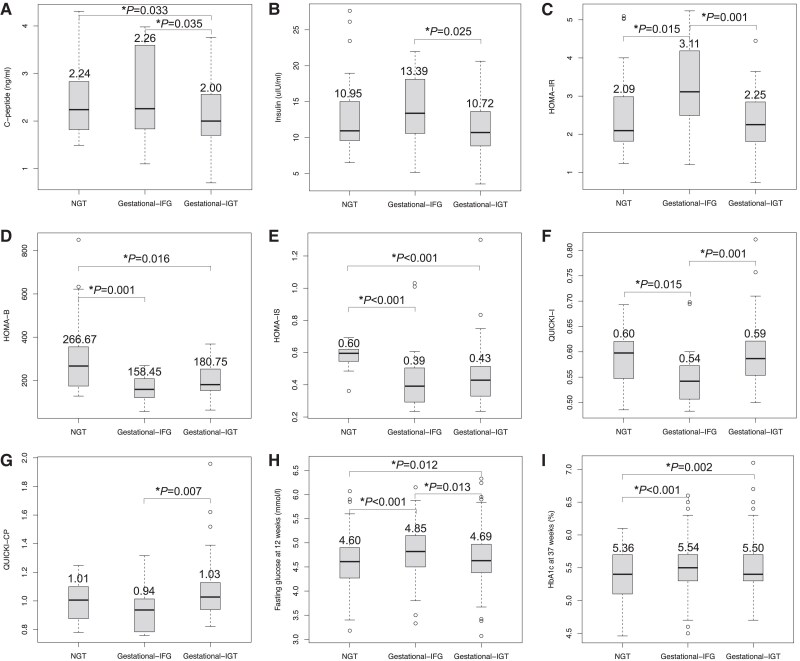
Parameters reflecting insulin physiology in NGT, gestational-IFG, and gestational-IGT groups.

There were 936 women in the NGT group, 378 women in the gestational-IFG group, and 736 women in the gestational-IGT group. Baseline characteristics of the 3 groups are shown in Table 1. Women in the NGT group were younger, less likely to have family history of diabetes, thinner at conception, and were more likely to be nulliparous. Maternal age, body weight, gravity, parity, FPG in the first trimester, and HbA1c at 37 weeks gestation increased across the NGT, gestational-IFG, and gestational-IGT groups. When comparisons were made between the gestational-IFG and gestational-IGT groups, women with gestational-IFG were younger (age 31.1 vs 31.6 years, *P* = .003), had a higher prepregnancy BMI (23.3 vs 22.6 kg/m^2^, *P* = .001), were less likely to be nulliparous (54.2% vs 66.2%, *P* < .001), more likely to have PCOS (17.7% vs 7.6%, *P* = .043), and had higher FPG in the first trimester (4.85 vs 4.69 mmol/L, *P* = .013) (Fig. 1H). The gestational-IFG group had greater need for insulin therapy compared to the gestational-IGT group (3.2% vs 1.1%, *P* = .021).

**Table 1.** Clinical characteristics of women with NGT, gestational-IFG, and gestational-IGT

	NGT (n = 936)	Gestational-IFG (n = 378)	Gestational-IGT (n = 736)	*P* value			
				Overall	NGT vs Gestational-IFG	NGT vs Gestational-IGT	Gestational-IFG vs Gestational-IGT
Age (years)	30.5 (30.2, 30.7)	31.1 (30.7, 31.5)	31.6 (31.4, 31.9)	<.001	.007	<.001	.003
Education				<.001	<.001	.048	.029
High school or lower	76 (8.1)	61 (16.1)	82 (11.1)				
College or higher	860 (91.9)	317 (83.9)	654 (88.9)				
Family history of diabetes	168 (17.9)	87 (23.1)	180 (24.4)	.191	.332	.100	.200
Drinking history	16 (1.7)	13 (3.5)	6 (0.8)	.443	.334	.476	.175
Smoking history	12 (1.3)	8 (2.1)	12 (1.6)	.864	.785	.660	.592
Gravity				.001	<.001	.027	.089
1-2	737 (78.7)	261 (69.1)	545 (74.1)				
≥ 3	199 (21.3)	117 (30.9)	191 (25.9)				
Parity				<.001	<.001	.149	<.001
0	638 (68.2)	205 (54.2)	487 (66.2)				
1	282 (30.1)	153 (40.5)	226 (30.7)				
2-3	16 (1.7)	20 (5.3)	23 (3.1)				
Polycystic ovary syndrome	59 (6.3)	67 (17.7)	56 (7.6)	.005	.001	.592	.043
Body weight							
Prepregnancy weight (kg)	56.6 (56.0, 57.1)	61.3 (60.4, 62.3)	59.1 (58.5, 59.8)	<.001	<.001	<.001	<.001
Prepregnancy BMI (kg/m^2^)	21.4 (21.2, 21.6)	23.3 (23.0, 23.7)	22.6 (22.4, 22.8)	<.001	<.001	<.001	.001
Weight at delivery (kg)	71.2 (70.6, 71.8)	74.8 (73.8, 75.9)	70.5 (69.8, 71.3)	<.001	<.001	.170	<.001
BMI at delivery (kg/m^2^)	27.0 (26.8, 27.2)	28.4 (28.1, 28.8)	27.0 (26.7, 27.2)	<.001	<.001	.231	<.001
Systolic blood pressure (mmHg)	112.1 (111.3, 113.1)	112.8 (110.1, 115.6)	117.4 (115.2, 119.6)	<.001	.662	<.001	.013
Diastolic blood pressure (mmHg)	70.4 (69.7, 71.1)	72.4 (69.9, 75.0)	73.0 (71.2, 74.6)	.032	.124	.006	.697
Fasting glucose in the first trimester (mmol/L)	4.60 (4.56, 4.64)	4.85 (4.74, 4.96)	4.69 (4.63, 4.76)	<.001	<.001	.012	.013
Insulin treatment	N/A	12 (3.2)	8 (1.1)	<.001	N/A	N/A	.021

Data are means (95% CI) for continuous variables and n (%) for categorial variables. Abbreviations: BMI, body mass index; IFG, impaired fasting glucose; IGT, impaired glucose tolerance; NGT, normoglycemia.

### Insulin Sensitivity and β-Cell Function

Compared with the NGT group, both gestational-IFG and gestational-IGT showed decreased β-cell function and insulin sensitivity. Women with gestational-IGT had deteriorated β-cell function, as assessed by lower C-peptide (2.00 vs 2.26 ng/mL, *P* = .035) and insulin concentrations (10.72 vs 13.39 µU/mL, *P* = .025) compared to those with gestational-IFG (Fig. 1A and 1B). HOMA-β were numerically lower in gestational-IFG than in gestational-IGT, although the difference was not statistically significant (158.45 vs 180.75, *P* = .058) (Fig. 1D). Women with gestational-IFG had greater insulin resistance (HOMA-IR 3.11 vs 2.25, *P* = .001) and decreased insulin sensitivity (HOMA-IS 0.39 vs 0.43, *P* = .001) (Fig. 1C and 1E). Results of QUICKI assessment also showed decreased insulin sensitivity in women with gestational-IFG (QUICKI-I 0.54 vs 0.59, QUICKI-CP 0.94 vs 1.03, both *P* <.01) compared to those with gestational-IGT (Fig. 1F and 1G).

### Pregnancy Outcomes

**Figure 2.**
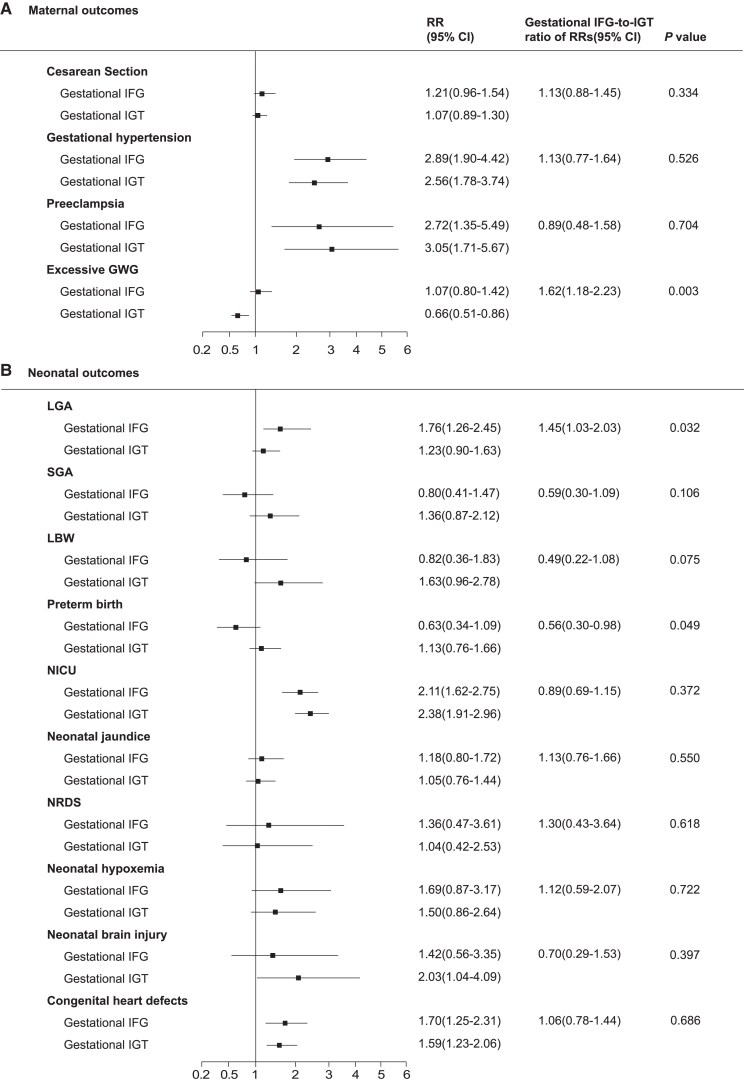
Association between GDM subtypes (gestational-IFG and gestational-IGT) and pregnancy outcomes-maternal outcomes (A), neonatal outcomes (B) in comparison with NGT. Abbreviations: GWG, gestational weight gain; LBW, low birth weight; LGA, large for gestational age; NICU, neonatal intensive care unit; NRDS, neonatal respiratory distress syndrome; SGA, small for gestational age.

The incidence of pregnancy outcomes in gestational-IFG and gestational-IGT groups are shown in Table 2. Both GDM subtypes had increased HbA1c levels in the third trimester (gestational-IFG 5.54%, and gestational-IGT 5.50%) compared with women in the NGT group (5.36%, both *P* <.01), whereas the difference was not significant between the gestational-IFG and gestational-IGT groups (*P* = .235) (Fig. 1I). Compared with NGT, similar patterns of increased risks of gestational hypertension, preeclampsia, NICU, and congenital heart defects were observed in both GDM subtypes, while gestational-IFG (but not gestational-IGT) was associated with a significantly higher risk of LGA (RR 1.76; 95% CI, 1.26-2.45; *P* < .001, Fig. 2). Neonatal brain injury was significantly increased in the gestational-IGT group (RR 2.03; 95% CI, 1.04-4.09; *P* = .041), but not in the gestational-IFG group (RR 1.42; 95% CI, 0.56-3.35; *P* = .439). Gestational-IGT showed nonsignificant but increased trend of SGA and LBW, whereas gestational-IFG exhibited a mitigated tendency, as shown in Fig. 2B. When compared to one another, gestational-IFG had a greater risk of excessive GWG (RR 1.62; 95% CI, 1.18-2.23; *P* = .003) (Fig. 2A) and LGA (RR 1.45; 95% CI, 1.03-2.03; *P* = .032) than did gestational-IGT. Gestational-IGT showed greater risks of preterm birth compared to gestational-IFG (RR 1.80; 95% CI, 1.02-3.36; *P* = .049).

**Table 2.** Maternal and neonatal outcomes of women with NGT, gestational-IFG, and gestational-IGT

	NGT group	Gestational-IFG group	Gestational-IGT group
	N = 936	N = 378	N = 736
**Maternal outcome**			
Cesarean delivery	445 (47.5)	198 (52.4)	363 (49.3)
Gestational hypertension	**46 (4.9)**	**49 (13.0)** *^a^*	**86 (11.7)** *^a^*
Preeclampsia	**16 (1.7)**	**17 (4.5)** *^a^*	**37 (5.0)** *^a^*
HbA1c at 37 gestational weeks (%)	**5.36 (5.28, 5.45)**	**5.54 (5.49, 5.59)** *^a^*	**5.50 (5.47, 5.53)** *^a^*
GWG (kg)	**14.5 (14.2, 14.9)**	**13.6 (13.0, 14.2)** *^a^*	**11.5 (11.1, 11.9)** *^a,b^*
Excessive GWG	**403 (43.1)**	**172 (45.5)**	**249 (33.8)** *^a,b^*
**Neonatal outcome**			
Gestation weeks at delivery (weeks)	**39.3 (39.2, 39.4)**	**39.2 (39.1, 39.4)**	**39.1 (39.0, 39.2)** *^a^*
Preterm birth	58 (6.2)	15 (4.0)	51 (6.9) *^b^*
Birth size			
Birth weight (kg)	**3.32** (**3.29, 3.35)**	**3.43 (3.38, 3.48)** *^a^*	**3.29 (3.25, 3.32)** *^b^*
Birth length (cm)	**50.1** (**50.0, 50.2)**	**50.4 (50.2, 50.5)** *^a^*	**50.0 (49.9, 50.1)** *^b^*
Z score (U)	0.7 (0.66, 0.73)	0.75 (0.68, 0.81)	0.7 (0.66, 0.75)
LGA	**104 (11.1)**	**68 (18.0)** *^a^*	**97 (13.2)** *^b^*
SGA	40 (4.3)	13 (3.5)	42 (5.7)
LBW	26 (2.8)	8 (2.1)	32 (4.4)
NICU	**193 (20.6)**	**134 (35.5)** *^a^*	**281 (38.2)** *^a^*
Neonatal jaundice	94 (10.0)	44 (11.7)	77 (10.5)
NRDS	11 (1.2)	6 (1.6)	9 (1.2)
Neonatal hypoxemia	24 (2.6)	16 (4.2)	28 (3.8)
Neonatal brain injury	14 (1.5)	7 (1.9)	22 (3.0) *^a^*
Congenital heart defects	**131 (14.0)**	**82 (21.7)** *^a^*	**152 (20.7)** *^a^*

Data are means (95% CI) for continuous variables and n (%) for categorial variables. Significant differences across the 3 groups are shown in bold.

Abbreviations: GWG, gestational weight gain; HbA1c, glycated hemoglobin; IFG, impaired fasting glucose; IGT, impaired glucose tolerance; LBW, low birth weight; LGA, large for gestational age; NGT, normoglycemia; NICU, neonatal intensive care unit; NRDS, neonatal respiratory distress syndrome; SGA, small for gestational age.

^a^ *P* < .05 in comparison with NGT group.

^b^ *P* < .05 in comparison with gestational-IFG group.

## Discussion

Our study had 3 major findings. First, women with GDM characterized by isolated IFG presented higher-risk clinical characteristics than those with IGT, in terms of higher BMI, a greater proportion of multiparity, and PCOS. In addition, women with IFG had higher requirement for insulin therapy than those with IGT. Second, gestational-IFG had greater insulin resistance and gestational-IGT had worse β-cell function when compared to one another. Third, gestational-IFG had relatively greater risks for excessive weight gain and LGA, whereas gestational-IGT had higher risk for preterm birth and neonatal brain injury.

The original definition for GDM, “any degree of glucose intolerance first detected during pregnancy,” encompassed overt GDM and pregestational diabetes. An early study that categorized GDM into subgroups based on body weight showed that the subgroup of maternal obesity had higher risk of macrosomia than the normal body weight group, while those with normal body weight exhibited deteriorated insulin secretion compared with those with obesity (16). Since the landmark Hyperglycemia and Adverse Pregnancy Outcome (HAPO) study proposed glucose thresholds for diagnosing GDM with OGTT, data on heterogeneity study of GDM have arisen and been inconsistent (17). A study in Canada which counted abnormalities in a glucose challenge test and an OGTT, concluded that there was a dose-response association between the number of abnormal glucose values and the risk of postpartum T2DM (18). An Israel mother-offspring cohort study further analyzed the future risk of offspring development in women who underwent two-step GDM screening, suggested that the number of glucose abnormalities during pregnancy predicted offspring overweight/obesity at late adolescence (19). Recently, the study group further elaborated on this issue regarding entire dysglycemia in pregnancy including cases that did not meet GDM criteria, found that women with fasting hyperglycemia had higher risk of developing postpartum T2DM compared to women with postload hyperglycemia (7). Research on pregnancy outcomes generally showed that the GDM subtype of fasting hyperglycemia exhibits a greater risk for LGA compared to those with postload hyperglycemia (5, 8-10). A population-based study concluded that fasting hyperglycemia had higher risk of gestational hypertension (aRR 1.51) than postload hyperglycemia (8); however, the grouping of fasting hyperglycemia did not consider postload level, making this group a potentially high-risk cluster encompass individuals with both fasting and postload hyperglycemia. In light of the extant literature, the heterogeneity of GDM warrants further discussion.

We previously reported more severe glucose fluctuations (higher time above target range) in pregnant women with IGT compared to women with IFG (13). The current study further investigated insulin physiology. Despite the concordant decrease in insulin sensitivity and β-cell function for both subtypes, gestational-IGT had even worse β-cell function manifest by lower level of C-peptide. Alongside our observation that women with gestational-IGT were thinner and older than women with gestational-IFG, β-cell dysfunction might be an essential element in the etiology of gestational-IGT, in contrast to gestational-IFG where hyperglycemia was predominantly ascribed to insulin resistance. We also observed that women with gestational-IFG had higher prepregnancy BMI and more likely to have PCOS, in accordance with previous reports (6, 11, 20). These findings align with the theory that adipose tissue plays an important role in hepatic insulin action, which dominates fasting glucose (21, 22). We suspect that insulin resistance is an essential issue in GDM specifically in individuals with overweight or PCOS. Women with fasting hyperglycemia and insulin resistance might experience failed suppression of endogenous glucose output and hyperinsulinemia, which contribute to maternal weight gain and excessive intrauterine fetal growth (23). In line with this, we found that the risks for excessive GWG and LGA were higher in the gestational-IFG group than in the gestational-IGT group. These findings have important clinical implications that strategies on weight management are beneficial for women with obesity or PCOS aiming to reduce the risk of GDM and excessive fetal growth.

The current study included HbA1c at 37 weeks gestation as a primary outcome, as HbA1c was found associated with obstetric outcomes and has been recommended by guidelines for research of diabetes in pregnancy (12, 24). We found that HbA1c level at 37 weeks gestation was lower in the gestational-IGT than in the gestational-IFG. Controversially, greater initiating of insulin therapy was found in gestational-IFG, similar to previous study (6). From one perspective, severe insulin resistance in gestational-IFG may pose a more challenging situation for treatment aiming to achieve normoglycemia during gestation. From another, we suspect that gestational-IGT might experience elevated but delayed insulin secretion after glucose or carbohydrate stimulation. This becomes a particularly significant issue concerning the prevention of hypoglycemia during insulin treatment for gestational-IGT.

A prospective study reported a greater incidence of preterm birth in IGT (11.3%) than in IFG (5.3%) (6). Similarly, a retrospective study showed an elevated risk of preterm birth in IGT (aOR 2.85) than in IFG (aOR 1.5) (11). The current study also found that babies born of gestational-IGT were at higher risk of preterm birth than those born of gestational-IFG. The mechanism of postload dysglycemia in connection with preterm birth is unclear, presumably associated with glucose fluctuations in the IGT or vascular inflammation. Essentially, maintaining glucose homeostasis after carbohydrate load and preventing preterm delivery would ideally be added to strategies of gestational-IGT management in clinical practice, and future studies are needed to reveal the physiology between gestational-IGT and preterm birth or to identify markers, if any, in the comorbidity of gestational-IGT and preterm birth. We assess various recommended core outcomes and serve as a complement to previous studies aiming to better understand the associations between GDM subtypes and outcomes. We found an increased trend of developing SGA and LBW for women with IGT, indicating that strategies to prevent insufficient birth weight should be implemented for women with postload hyperglycemia. Notably, there was a significant risk ratio for neonatal brain injury in gestational-IGT. The mechanism of the association between maternal hyperglycemia and fetal brain tissue was illustrated in a recently published study (25). The author reported altered amino acid neurotransmitter levels in the fetal brain of pregnant Akita mice (a mode of diabetes caused by insulin dysfunction), indicating that there might be a link between diabetes triggered by insulin deficiency and congenital brain defects.

Our study has several strengths. First, this study provides data on insulin physiology (insulin resistance/sensitivity, β-cell function) of GDM subtypes, and we evaluated the associations with multiple core pregnancy outcomes that were rigorously recorded. Second, detailed information regarding maternal prepregnancy weight, family history of diabetes, parity, and glucose parameters from early to late pregnancy were collected. Confounding variables were therefore controlled in the analysis. Third, our study consistently followed a standard protocol for the management of GDM, and potential bias was minimized. However, some limitations need to be acknowledged. First, women were mostly enrolled after conception. As such, we could not explicitly exclude those who had pregestational glucose intolerance or capture metabolic profiles before pregnancy. Second, the follow-up period was short, limiting the observation of developing postpartum T2DM. Third, we did not measure C-peptide and insulin level under postload conditions, which could have provided insight into insulin action after glucose stimulation. Fourth, while HOMA has been proven to be a reliable algebraic index for estimating insulin physiology in general population, its reliability is limited in pregnant populations due to the complexities of glucose metabolism during pregnancy. Nevertheless, HOMA can still provide some insights into the differences in insulin physiology across groups.

In conclusion, our findings suggest that GDM characterized by isolated fasting hyperglycemia was associated with increased insulin resistance and increased risks of excessive weight gain and LGA, whereas isolated postload hyperglycemia was associated with deficient β-cell function and confers higher risks of preterm birth and neonatal brain injury compared to isolated fasting hyperglycemia. Our findings provide evidence of diverse strategies regarding various physiologies and outcomes of GDM subtypes.

## Data Availability

The data underlying this article can be shared on reasonable request to the corresponding author.

## Abbreviations

BMI: body mass index
DBP: diastolic blood pressure
FPG: fasting plasma glucose
GDM: gestational diabetes mellitus
gestational-IFG: GDM characterized by isolated impaired fasting glucose
gestational-IGT: GDM characterized by isolated impaired postload glucose tolerance
GWG: gestational weight gain
HbA1c: glycated hemoglobin
HOMA-B: homeostatic model assessment of beta-cell function
HOMA-IR: homeostatic model assessment of insulin resistance
HOMA-IS: homeostatic model assessment of insulin sensitivity
IFG: impaired fasting glucose
IGT: impaired glucose tolerance
LBW: low birth weight
LGA: large for gestational age
NGT: normoglycemia
NICU: neonatal intensive care unit
NRDS: neonatal respiratory distress syndrome
OGTT: oral glucose tolerance test
PCOS: polycystic ovary syndrome
QUICKI-CP: quantitative insulin sensitivity check index calculated with C-peptide
QUICKI-I: quantitative insulin sensitivity check index calculated with insulin
RR: risk ratio
SBP: systolic blood pressure
SGA: small for gestational age
T2DM: type 2 diabetes mellitus
